# The Vienna psychosocial assessment procedure for bionic reconstruction in patients with global brachial plexus injuries

**DOI:** 10.1371/journal.pone.0189592

**Published:** 2018-01-03

**Authors:** Laura Antonia Hruby, Anna Pittermann, Agnes Sturma, Oskar Christian Aszmann

**Affiliations:** 1 Christian Doppler Laboratory for Restoration of Extremity Function, Division of Plastic and Reconstructive Surgery, Department of Surgery, Medical University of Vienna, Vienna, Austria; 2 General Hospital of Vienna, Department of Clinical Psychology, Vienna, Austria; 3 Division of Plastic and Reconstructive Surgery, Department of Surgery, Medical University of Vienna, Vienna, Austria; 4 Health Assisting Engineering, University of Applied Sciences FH Campus, Vienna, Austria; BG Trauma Center Ludwigshafen, GERMANY

## Abstract

**Background:**

Global brachial plexopathies cause major sensory and motor deficits in the affected arm and hand. Many patients report of psychosocial consequences including chronic pain, decreased self-sufficiency, and poor body image. Bionic reconstruction, which includes the amputation and prosthetic replacement of the functionless limb, has been shown to restore hand function in patients where classic reconstructions have failed. Patient selection and psychological evaluation before such a life-changing procedure are crucial for optimal functional outcomes. In this paper we describe a psychosocial assessment procedure for bionic reconstruction in patients with complete brachial plexopathies and present psychosocial outcome variables associated with bionic reconstruction.

**Methods:**

Between 2013 and 2017 psychosocial assessments were performed in eight patients with global brachial plexopathies. We conducted semi-structured interviews exploring the psychosocial adjustment related to the accident, the overall psychosocial status, as well as motivational aspects related to an anticipated amputation and expectations of functional prosthetic outcome. During the interview patients were asked to respond freely. Their answers were transcribed verbatim by the interviewer and analyzed afterwards on the basis of a pre-defined item scoring system. The interview was augmented by quantitative evaluation of self-reported mental health and social functioning (SF-36 Health Survey), body image (FKB-20) and deafferentation pain (VAS). Additionally, psychosocial outcome variables were presented for seven patients before and after bionic reconstruction.

**Results:**

Qualitative data revealed several psychological stressors with long-term negative effects on patients with complete brachial plexopathies. 88% of patients felt functionally limited to a great extent due to their disability, and all of them reported constant, debilitating pain in the deafferented hand. After bionic reconstruction the physical component summary scale increased from 30.80 ± 5.31 to 37.37 ± 8.41 (p-value = 0.028), the mental component summary scale improved from 43.19 ± 8.32 to 54.76 ± 6.78 (p-value = 0.018). VAS scores indicative of deafferentation pain improved from 7.8 to 5.6 after prosthetic hand replacement (p-value = 0.018). Negative body evaluation improved from 60.71 ± 12.12 to 53.29 ± 11.03 (p-value = 0.075). Vital body dynamics increased from 38.57 ± 13.44 to 44.43 ± 16.15 (p-value = 0.109).

**Conclusions:**

Bionic reconstruction provides hope for patients with complete brachial plexopathies who have lived without hand function for years or even decades. Critical patient selection is crucial and the psychosocial assessment procedure including a semi-structured interview helps identify unresolved psychological issues, which could preclude or delay bionic reconstruction. Bionic reconstruction improves overall quality of life, restores an intact self-image and reduces deafferentation pain.

## Introduction

Brachial plexus injuries (BPI) including avulsion of multiple nerve roots represent a severe neurological condition with major life-long functional and psychosocial consequences [1],[2],[3]. Traumatic nerve root avulsions are characteristic of young adults who have been involved in motorcycle accidents [4],[5]. Complete brachial plexus lesions result in loss of arm and hand function [6], anesthesia and missing sensory protection [7], and—in most cases of avulsion injuries—severe chronic deafferentation pain [8]. The continuous increase in the number of civilian brachial plexus lesions due to high-velocity traumata has promoted tremendous progress in surgical techniques for brachial plexus repair [9]. Stabilization of the shoulder joint and restoration of elbow function can be achieved in the majority of patients, however, in global plexopathies with lower root avulsions hand function remains negligible [2],[10],[11].

### Psychosocial consequences of brachial plexus injury

Despite the fact that some global extremity function can be restored in these grave situations, patients still suffer a great deal of stress which can hinder psychosocial adjustment following the accident [12]. Objectively good surgical outcomes do not always meet patients’ expectations and/or functional needs and thus do not automatically translate into psychological wellbeing [13]. E.g., restoration of finger flexion or wrist extension scoring M3 is not much appreciated and patients still prefer thoracobrachial and abdominal grasping methods for holding objects [9]. Affected patients often still have to cope with marked pain and specific hand/arm disabilities, which restricts important daily life activities [14]. Sources of stress include chronic deafferentation pain, functional disability, unemployment, financial instability and dissatisfaction with aesthetic appearance [12]. The inability to return to work greatly affects psychological well being, the patients’ relationships and, ultimately, their independence in every day life [15]. In patients with complete BPIs Kretschmer et al. reported symptoms of depression and/or anxiety in 28%, further showing that occupational retraining was highest among the complete lesion group with 45% of patients incapacitated for work or unemployed even after retraining [14].

In longstanding brachial plexus avulsion injuries and prolonged denervation times, the extremity eventually becomes atrophic and cold with a distal bluish discoloration due to autonomic paralysis [16]. Patients living with such an apparent physical deficit cope with increased psychological distress and have to compensate for potential social pressure [17]. Conspicuous differences in physical appearance may result in shame and trigger a range of concealing behaviors in response to a disturbed body image and negative self-evaluation [17]. Finally, due to the fact that these hands and arms are insensate and paralyzed they are often subject to inadvertent injury and need to be protected in a sling.

### Bionic reconstruction

**Fig 1:**
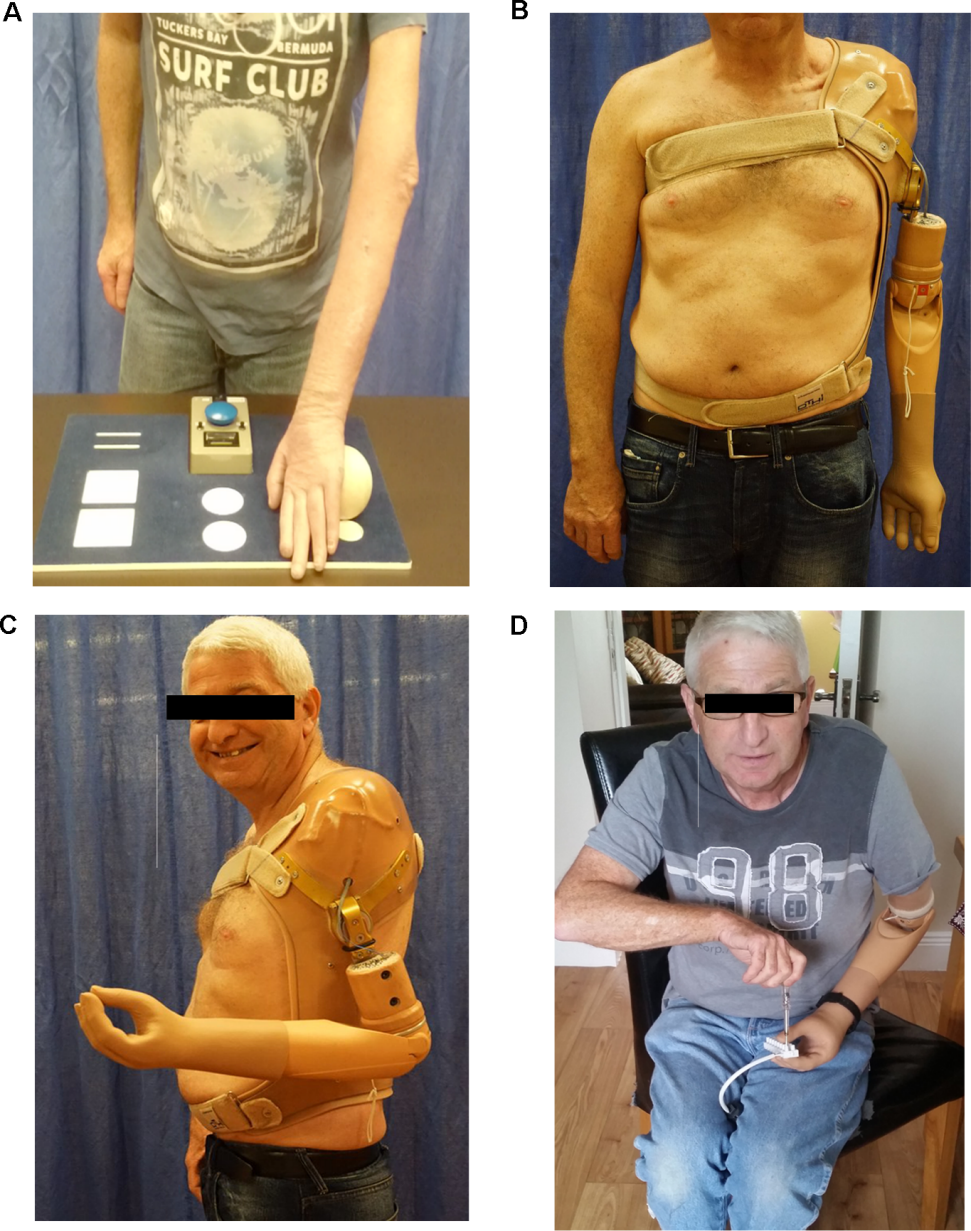
Bionic reconstruction in a patient with complete BPI. **A**, A 54-year old patient with a flail, atrophic left arm following a BP avulsion injury with a denervation time of 7,5 years. Picking up a small light-weight ball during functional testing is impossible with the functionless hand. **B**, Prosthetic arm replacement after elective amputation of the left arm at glenohumeral level. **C**, The prosthetic arm is driven by two electromyographic signals, which are intuitively controlled by the patient (Cutaneous EMG-sensor position on infraspinatus muscle and pectoralis major muscle). **D**, Prosthetic usage in daily life activities. *BP*, *brachial plexus*. *BPI*, *brachial plexus injury*.

In severe cases of BPI, where primary and secondary reconstruction procedures have failed to improve arm and hand function, a novel treatment approach has recently been described to restore hand function in the first three patients [18]. *Bionic reconstruction* includes a combination of selective nerve and/or muscle transfers to establish electromyographic (EMG) signal sites in the fore- und upper arm which, after elective amputation of the functionless limb, allows the control of a myoelectric prosthetic hand [18] (Fig 1). Patient selection, however, is crucial in the context of bionic reconstruction, since the amputation of a limb–even if without functional use to the patient–represents an irreversible, life changing procedure. Similar to other elective interventions (such as bariatric surgery, gender reassignment, aesthetic surgeries, etc.), the elective amputation of a limb necessitates the performance and documentation of a psychological assessment prior to the surgery. Additionally patients need to be aware that intensive rehabilitation programs are essential after potential nerve surgery as well as for optimal future prosthetic control subsequent to amputation [19].

Although studies are available which have analyzed functional outcome measures after bionic hand reconstruction in the first five patients including objective hand function tests [18],[19], none of these have thoroughly addressed the needs of a standardized psychosocial evaluation prior to the irreversible decision of an elective amputation. Since psychosocial variables, coping and post-injury adjustment are difficult to evaluate using quantitative methods due to individual beliefs and emotions [12], we chose to take a qualitative approach, augmented by three validated, patient-reported questionnaires, which added to the evaluation of the overall psychosocial status of a patient.

We therefore introduce the “Vienna psychosocial assessment procedure for bionic reconstruction”, which includes a semi-structured interview as well as three patient-reported questionnaires. The interview was developed and conducted by one of the authors (A.P.), addressing psychosocial adjustment after global brachial plexus injury, psychosocial status of the patient, and motivational aspects and expectations related to an anticipated amputation and prosthetic replacement of the functionless hand. The self-reported questionnaires evaluate deafferentation pain, quality of life and body image. The primary purpose of this paper was to present our assessment (including the interview and the questionnaires), which evaluates a patient’s overall psychosocial status, identifies a patient’s motivations and expectations related to elective amputation and uncovers unresolved psychological issues, which could delay or preclude successful bionic reconstruction. The second purpose of this paper was to present psychosocial outcome variables including changes in chronic pain, overall quality of life and body image evaluation in seven patients and to discuss effects of bionic hand reconstruction on these parameters.

## Methods

### Patients

Between 2013 and 2017, eight patients with global BPIs were psychosocially evaluated for bionic hand reconstruction at the Christian Doppler Laboratory for Restoration of Extremity Function.

In all patients multiple nerve root avulsions were present and primary and secondary reconstructive procedures have failed to improve arm and hand function. In 6 patients (75%) motorcycle accidents caused the BPI, the remaining two suffered a skiing accident. All patients were male. The mean age at evaluation for bionic hand reconstruction was 45 ± 17 years (range, 23–76) with a mean denervation time of 8 ± 7 years (range, 1 ± 21). The mean follow-up period was 10 ± 6 months (range, 3–17). Table 1 briefly summarizes the myosignals of each patient used for prosthetic control.

**Table 1:** EMG signals in all patients used for prosthetic control.

Patient ID	EMG signals in the fore- and upper arm at initial consultation	Surgeries to improve the biotechnological interface and/or to create additional EMG signal sites
**1**	biceps muscle + triceps muscle	free gracilis muscle transfered to medial upper arm and neurotization of median nerve to obturator nerve to generate a third EMG signal
**2**	pronator teres muscle + forearm extensor compartment	ND
**3**	biceps muscle + triceps muscle	transfer of pedicled biceps and triceps muscles to supraclavicular fossa and supraspinatous fossa to facilitate prosthetic fitting
**4**	infraspinatus muscle + pectoralis major muscle	ND
**5**	biceps muscle + pectoralis major muscle + brachioradialis muscle	transfer of pedicled brachioradialis muscle to upper arm to preserve this signal site upon elective transhumeral amputation
**6**	pronator teres muscle + forearm extensor compartment	ND
**7**	two separable signals at the forearm extensor compartment	ND
**8**	supraspinatus muscle + pectoralis major muscle	ND

At initial consultation residual muscle activity is assessed in the fore- and upper arm using surface EMG electrodes. At least two myosignals are needed for reliable prosthetic control. In some patients surgery was performed to improve the future biotechnological interface and/or to create additional EMG signal sites. *EMG*, *electromyographic; ND*, *not done*.

The Institutional Review Board of the Medical University of Vienna approved all aspects of this procedure. Patients gave written informed consent. The patient in Fig 1 has given written informed consent (as outlined in PLOS consent form) to publish these case details, i.e. photographs.

### The “Vienna psychosocial assessment procedure”

**Fig 2:**
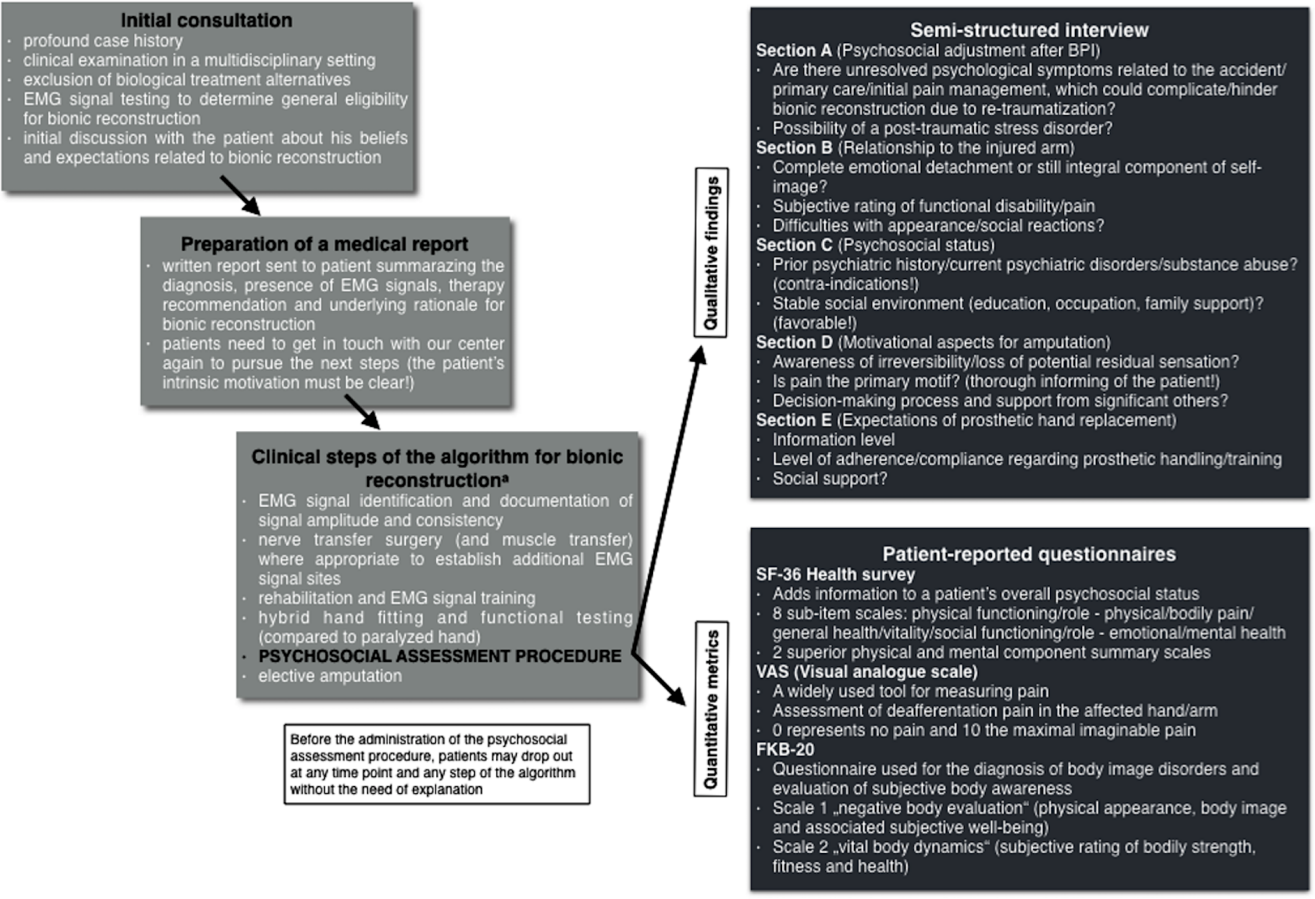
Scheme illustrating the „Vienna psychosocial assessment procedure“. The assessment procedure is an integral component of the treatment algorithm for bionic reconstruction in patients with complete brachial plexus injury. The pre-surgical psychosocial assessment prior to elective amputation includes qualitative findings obtained from a semi-structured interview and quantitative metrics obtained from three questionnaires. The intended purpose of this assessment was to evaluate the overall psychosocial status of a patient, to uncover psychological disorders which would preclude successful prosthetic reconstruction and to offer psychological support where appropriate. EMG, electromyographic. a The complete treatment algorithm for bionic reconstruction ranging from identification of eligible patients to final prosthetic fitting can be found here: [19].

The psychosocial assessment procedure (including the semi-structured interview and three patient-reported questionnaires) is an integral component of the treatment algorithm for bionic reconstruction [19] and is administered 1 month to 1 week prior to amputation depending on whether patients live in/near Vienna or come to Vienna shortly before surgery (i.e., amputation). The assessment is performed only in patients who have successfully completed the preceding steps of the treatment algorithm for bionic reconstruction (see Fig 2) with the intended purpose to identify patients who

- do not qualify for bionic reconstruction due to unresolved psychological issues (e.g., post-traumatic stress disorder, substance abuse, prior psychiatric history), due to unfavorable motives (e.g., pain being the primary reason for amputation, unawareness of irreversibility) and/or unrealistic expectations of prosthetic reconstruction (e.g., over-estimation of prosthetic function, low compliance or adherence);
- need psychological support to overcome minor psychological issues in order to proceed with the procedure of bionic reconstruction (support is also offered to family members where appropriate);
- need further information as to what can be expected from prosthetic reconstruction (e.g., organization of a get-together with a patient who has already undergone bionic reconstruction).

The operating surgeon is not involved in the psychosocial assessment and is not accountable for deciding whether a patient is psychosocially capable of undergoing prosthetic reconstruction. Instead, this decision is made in synopsis with the multidisciplinary team after the psychologist has conducted the psychosocial evaluation. If a patient’s psychosocial status and/or his motives represent exclusion criteria for bionic reconstruction these are transparently disclosed to each patient in detail in a separate conversation.

### Semi-structured interview

We followed a primarily qualitative approach, using a semi-structured interview to explore patients’ psychological, social, and emotional experiences after global BPI and their current relationship to the affected arm, overall psychosocial status, as well as expectations and motivational aspects for an elective amputation and prosthetic replacement of the functionless limb. As such the interview was divided into sections of questions relating to five different categories, A to E. A clinical psychologist (A.P.) developed its structure, content and analysis and conducted all interviews for consistency. Patients were asked to respond freely. Their answers were transcribed verbatim by the interviewer and analyzed afterwards on the basis of a pre-defined item scoring system (see data analysis). The duration of interview was 90 to 120 minutes.

### Data analysis

Each of the five interview sections (A-E) consisted of 5 to 9 different items of potential psychological symptomatology (Table 2). Each item was assigned a value of 0 or 1 with 0 indicating no problem in the addressed domain and 1 indicating the presence of risk factors and/or psychological stress in the addressed domain. Taking for example item E1 (“Information level of prosthetic hands”), statements such as “I have had the opportunity to talk to a prosthetic user” or “I have trained with a hybrid prosthetic hand before” are rated with 0 as this implies good information level. Statements such as “I have seen some cool videos on Youtube but have never seen a prosthesis in real life”, on the contrary, are rated with 1 since there is potential risk that the patient does not know what to expect from a prosthetic hand. In this case additional information would be provided to ensure realistic expectations.

**Table 2:** Psychosocial topics of the semi-structured interview for the assessment of candidates for bionic hand reconstruction.

SCALE	Category	Items	Scale score (range)
A	Psychosocial adjustment after BPI	A1. Psychologically traumatizing accident	0 to 6
		A2. Debilitating circumstances related to the accident	
		A3. Symptoms of posttraumatic stress disorder	
		A4. Subjective perception of primary care	
		A5. Resources and stress management at time of accident	
		A6. Pain management following the accident	
B	Relationship to the injured arm/ Self-Perception of the injured arm	B1. Experience of functional disability	0 to 5
		B2. Pain now	
		B3. Worries and difficulties with physical appearance	
		B4. Experience of injured arm as belonging to self (neglect)	
		B5. Difficulties with social reactions	
C	Psychosocial status	C1. Professional education	0 to 9
		C2. Work now	
		C3. Work in the future (with prosthesis)	
		C4. Social resources/quality of family support	
		C5. Pain medication	
		C6. Alcohol	
		C7. Substance abuse	
		C8. Prior psychiatric history	
		C9. Coping strategies	
D	Motivational aspects related to an anticipated amputation	D1. Decision-making process	0 to 5
		D2. Awareness of the loss of potential residual sensation	
		D3. Awareness regarding the irreversibility of decision	
		D4. Awareness of the fact that deafferentation pain will not be cured by amputation–Pain is not the primary reason for amputation	
		D5. Support for decision of amputation by significant others	
E	Prosthetic fitting	E1. Information level about prosthetic hands	0 to 5
		E2. Awareness of functional limitations of a prosthetic hand	
		E3. Adherence level regarding difficulties with the prosthetic hand (mechanical defects, socket design and sensor position, etc.)	
		E4. Level of compliance regarding instructions in handling the prosthetic device (swimming, showering, etc.) and training demands	
		E5. Social Reactions to prosthetic device	
		MAXIMUM SCORE	30

Each scale consists of 5 to 9 items, each with a value of 0 or 1, adding up to a maximum score of 30.

Values of the items added up to sum scores of scales A to E between 0 and 9 depending on the number of items in the section. The minimum total score was 0 and the maximum total score 30. This approach of data analysis allowed intra-individual comparison of results as well as inter-individual comparison of scores among different patients.

**Section A** (Score 0–6) involved psychosocial adjustment, coping strategies and traumatic reactions related to the accident that has led to the BPI. In various studies brachial plexus injury has been found to entail psychosocial consequences in a considerable subset of patients [12–15, 20]. The identification of unresolved psychological sequelae (such as the possibility of a posttraumatic stress disorder) in this interview section would indicate inadequate psychosocial adjustment following the accident. In such patients, the prospect of elective amputation is considered inauspicious due to the possibility of aggravating already present psychological symptoms.

**Section B** (Score 0–5) covered the affective relationship of the patient to the injured arm including functional limitations resulting from the disability, pain, problems with disfigurement, body image issues and social reactions. These items were included to document a patient’s finalized persuasion that his arm or hand does no longer add to an intact self-image due to the absent nerval input connecting it to his brain. Incomplete emotional detachment from the hand would indicate that a patient does not (yet) qualify for bionic reconstruction.

**Section C** (Score 0–9) addressed the overall psychosocial status of the patient including possible risk factors and positive resources. Information on education, employment status, quality of relationships, substance abuse and prior psychiatric history were retrieved. Drawing on our experience a stable social environment (regular occupation, supportive family/friends, fair education status) is considered important in the context of bionic reconstruction. Psychiatric disorders (including substance abuse) on the other hand are defined contraindications for bionic reconstruction.

**Section D** (Score 0–5) involved motivational aspects related to the anticipated elective amputation of the injured hand. Questions covered the decision making process of the patient, the information level and social support guiding the decision as well as outcome expectations. The patient’s intrinsic motivation to pursue prosthetic reconstruction and awareness of the irreversibility of the decision need to be confirmed in this part of the interview.

**Section E** (Score 0–5) addressed the patient’s expectations related to the replacement of the functionless hand with a prosthetic device. Information level and knowledge about limitations of the prosthetic hand, compliance, and social reactions were rated. Unless the patient has realistic expectations he is provided additional information. Delusive expectations of prosthetic function are considered contra-indications for bionic reconstruction.

After this qualitative approach, our assessment procedure was supplemented with three questionnaires addressing overall quality of life (QoL, SF-36 Health Survey), body image issues (FKB-20) and deafferentation pain (visual analogue scale, VAS). These quantitative metrics added to the overall psychosocial status of the patient prior to reconstruction. Since this data was assessed twice (during the psychosocial assessment procedure *before* surgery and *after* successful bionic hand reconstruction) the impact of bionic reconstruction on these psychosocial variables could be objectively analyzed over time.

### SF-36 Health Survey

Patients’ QoL was evaluated with the SF-36 Health Survey (4-week recall) [21].

The questionnaire addresses 8 independent subscales: physical functioning, role—physical, bodily pain, general health, vitality, social functioning, role—emotional, and mental health. Based on these subscales, two superior physical and mental component summary scales can be identified [21]. All subscales and summary scales have mean values of 50 with a standard deviation of 10, with calculated T-value scores above 60 indicating above average health and scores below 40 indicating below average health compared against published age- and sex-matched norm samples.

### Body image questionnaire (FKB-20)

The FKB-20 is a 20-item questionnaire used for the diagnosis of body image disorders and evaluation of subjective body awareness [22]. Based on the questions two independent scales can be identified, negative body evaluation and vital body dynamics. The scale negative body evaluation includes physical appearance, body image and associated subjective well-being with a person’s body image [23]. The scale vital body dynamics describes how bodily strength, fitness and health are subjectively rated by the patient [23].

### Deafferentation pain

The patients’ pain levels in the hand and arm were assessed with the visual analog scale (VAS), a widely used tool for measuring pain by asking the patient to mark his or her perceived pain intensity with a point along a 10-cm horizontal line, whereby a score of 0 represents no pain and 10 the maximal imaginable pain [24].

### Statistical analysis

Data are presented either in absolute and relative values or as means and standard deviations. Differences in quality of life (SF-36 Health Survey data), body image disturbances (FKB-20) and VAS scores were analyzed using a paired, two-tailed Mann-Whitney-U-Test due to non-normalized distribution of data. A p-value < 0,05 was considered to be significant. The statistical analysis was performed using SPSS 22 (IBM Statistics, USA).

## Results

### Semi-structured interview

The number of patients scoring 1 for each interview item is listed in Table 3. A score of 1 indicated the presence of risk factors and/or psychological stress in the addressed item.

**Table 3:** Interview item response data.

SCALE	Category	Interview items	Number of patients n (%) scoring 1
A	Psychosocial adjustment after BPI	A1. Psychologically traumatizing accident	1 (12,5%)
		A2. Debilitating circumstances related to the accident	3 (37,5%)
		A3. Symptoms of posttraumatic stress disorder	0 (0%)
		A4. Subjective perception of primary care	3 (37,5%)
		A5. Resources and stress management at time of accident	0 (0%)
		A6. Pain management following the accident	7 (87,5%)
B	Relationship to the injured arm/ Self-Perception of the injured arm	B1. Experience of functional disability	1 (12,5%)
		B2. Pain now	8 (100%)
		B3. Worries and difficulties with physical appearance	0 (0%)
		B4. Experience of injured arm as belonging to self (neglect)	3 (37,5%)
		B5. Difficulties with social reactions	0 (0%)
C	Psychosocial status	C1. Professional education	0 (0%)
		C2. Work now	3 (37,5%)
		C3. Work in the future (with prosthesis)	1 (12,5%)
		C4. Social resources/quality of family support	0 (0%)
		C5. Pain medication	8 (100%)
		C6. Alcohol	0 (0%)
		C7. Substance abuse	1 (12,5%)
		C8. Prior psychiatric history	0 (0%)
		C9. Coping strategies	1 (12,5%)
D	Motivational aspects related to an anticipated amputation	D1. Decision-making process	0 (0%)
		D2. Awareness of the loss of potential residual sensation	0 (0%)
		D3. Awareness regarding the irreversibility of decision	0 (0%)
		D4. Awareness of the fact that deafferentation pain will not be cured by amputation–Pain is not the primary reason for amputation	1 (12,5%)
		D5. Support for decision of amputation by significant others	3 (37,5%)
E	Prosthetic fitting	E1. Information level about prosthetic hands	1 (12,5%)
		E2. Awareness of functional limitations of a prosthetic hand	0 (0%)
		E3. Adherence level regarding difficulties with the prosthetic hand (mechanical defects, socket design and sensor position, etc.)	0 (0%)
		E4. Level of compliance regarding instructions in handling the prosthetic device (swimming, showering, etc.) and training demands	0 (0%)
		E5. Social Reactions to prosthetic device	2 (25%)

A score of 1 for each item indicated the presence of risk factors and/or psychological issues, which could preclude or delay bionic reconstruction. The number of subjects (%) scoring 1 is shown for each item.

In interview section A seven of eight patients (88%) reported inadequate pain management during primary care shortly after the accident. In none of the patients symptoms of a posttraumatic stress disorder were encountered at the time of interview. In interview section B only one patient reported not to feel functionally limited by his injury, which was scored with 1 as the purpose of an elective amputation and bionic reconstruction is to improve function. All eight patients (100%) reported constant, chronic pain in the hand. Again in interview section C, the most encountered stressor in all patients was constant pain, however, all of them were satisfied with their social environment and support related to their disability and their pain. In section D only three patients (38%) reported problematic motives, mostly because they feared ambiguous reactions to an amputation from their social environment. In interview section E (prosthetic replacement of the hand) most patients (88%) had a good information level about prosthetic hands. Two patients (25%) feared ambiguous social reactions to a prosthetic device.

### SF-36 Health Survey

As only seven of eight patients had actually undergone bionic reconstruction, pre- and post-interventional data on QoL (SF-36 Health Survey), body image (FKB-20) and deafferentation pain are presented for seven patients (Case No. 8 has been evaluated for candidacy of bionic hand reconstruction but has not yet initiated the process).

Upon initial evaluation the mean scores of all subscales (physical functioning, role—physical, bodily pain, vitality social functioning, role—emotional, mental health) except for general health were below average (Table 4). After elective amputation and bionic hand reconstruction a significant improvement of quality of life was observed in the following subscales: physical functioning (p-value = 0.028), role—physical (p-value = 0.028), bodily pain (p-value = 0.041), vitality (p-value = 0.018), social functioning (p-value = 0.027), and mental health (p-value = 0.018). The physical component summary scale increased from 30.80 ± 5.31 to 37.37 ± 8.41 (p-value = 0.028), and the mental component summary scale improved from 43.19 ± 8.32 to 54.76 ± 6.78 (p-value = 0.018).

**Table 4:** SF-36 Health Survey data at initial evaluation and after bionic reconstruction.

	Case No.	Physical Functioning	Role–Physical	Bodily Pain	General Health	Vitality	Social Funtioning	Role–Emotional	Mental Health
**INITIAL EVALUATION**	1	28	10	18	56	38	32	36	48
	2	36	20	24	50	30	20	53	40
	3	39	29	34	54	44	48	39	52
	4	28	22	34	17	26	10	28	20
	5	0	0	14	38	24	24	18	35
	6	48	22	37	50	32	26	54	40
	7	0	15	17	12	34	16	10	32
	**MEAN± SD**	25.57 ± 18.76	16.86 ± 9.53	25.43 ± 9.48	39.57 ± 18.11	32.57 ± 6.90	25.14 ± 12.32	34.00 ± 16.62	38.14 ± 10.59
**AFTER** **BIONIC HAND RECONSTRUCTION**	1	44	52	33	56	40	54	53	52
	2	42	29	30	48	46	34	25	52
	3	44	54	37	56	56	55	53	62
	4	40	22	37	20	32	26	54	42
	5	14	52	33	44	48	46	53	60
	6	46	39	34	44	36	26	54	42
	7	4	44	36	6	41	47	53	58
	**MEAN± SD**	33.43 ± 17.04	41.71 ± 12.42	34.29 ± 2.56	39.14 ± 18.97	42.71 ± 8.01	41.14 ± 12.42	49.29 ± 10.72	52.57 ± 8.14
**Mann Whitney-U test**		0.028	0.028	0.041	0.823	0.018	0.027	0.173	0.018

### Body image questionnaire (FKB-20)

Our patients showed diverse results regarding their body image evaluation (Table 5). During initial evaluation more than half reported a massive negative body evaluation and reduced values on the FKB-20 scale vital body dynamics, which correlated with social withdrawal from leisure activities mostly due to chronic pain experienced by the patients. After final prosthetic hand replacement negative body evaluation improved from an above average mean value of 60.71 ± 12.12 to an average mean value of 53.29 ± 11.03 (p-value = 0.075). Vital body dynamics increased from 38.57 ± 13.44 to 44.43 ± 16.15 (p-value = 0.109).

**Table 5:** FKB-20 body image questionnaire data.

	Case No.	Negative body evaluation	Vital body dynamics
**INITIAL EVALUATION**	1	73^AA^	27^BA^
	2	73^AA^	37^BA^
	3	45^A^	63^AA^
	4	53^A^	27^BA^
	5	58^A^	47^A^
	6	50^A^	27^BA^
	7	73^AA^	42^A^
	**MEAN± SD**	60.71 ± 12.12	38.57 ± 13.44
**AFTER BIONIC HAND RECONSTRUCTION**	1	53^A^	47^A^
	2	73^AA^	37^BA^
	3	37^BA^	73^AA^
	4	50^A^	27^BA^
	5	47^A^	47^A^
	6	55^A^	27^BA^
	7	58^A^	53^A^
	**MEAN± SD**	53.29 ± 11.03	44.43 ± 16.15
**Mann-Whitney U-Test**		0.075	0.109

Values above 60 or below 40 indicate pathological results that diverge from a norm sample of healthy subjects.

AA, above average.

A, average.

BA, below average.

### Deafferentation pain

**Fig 3:**
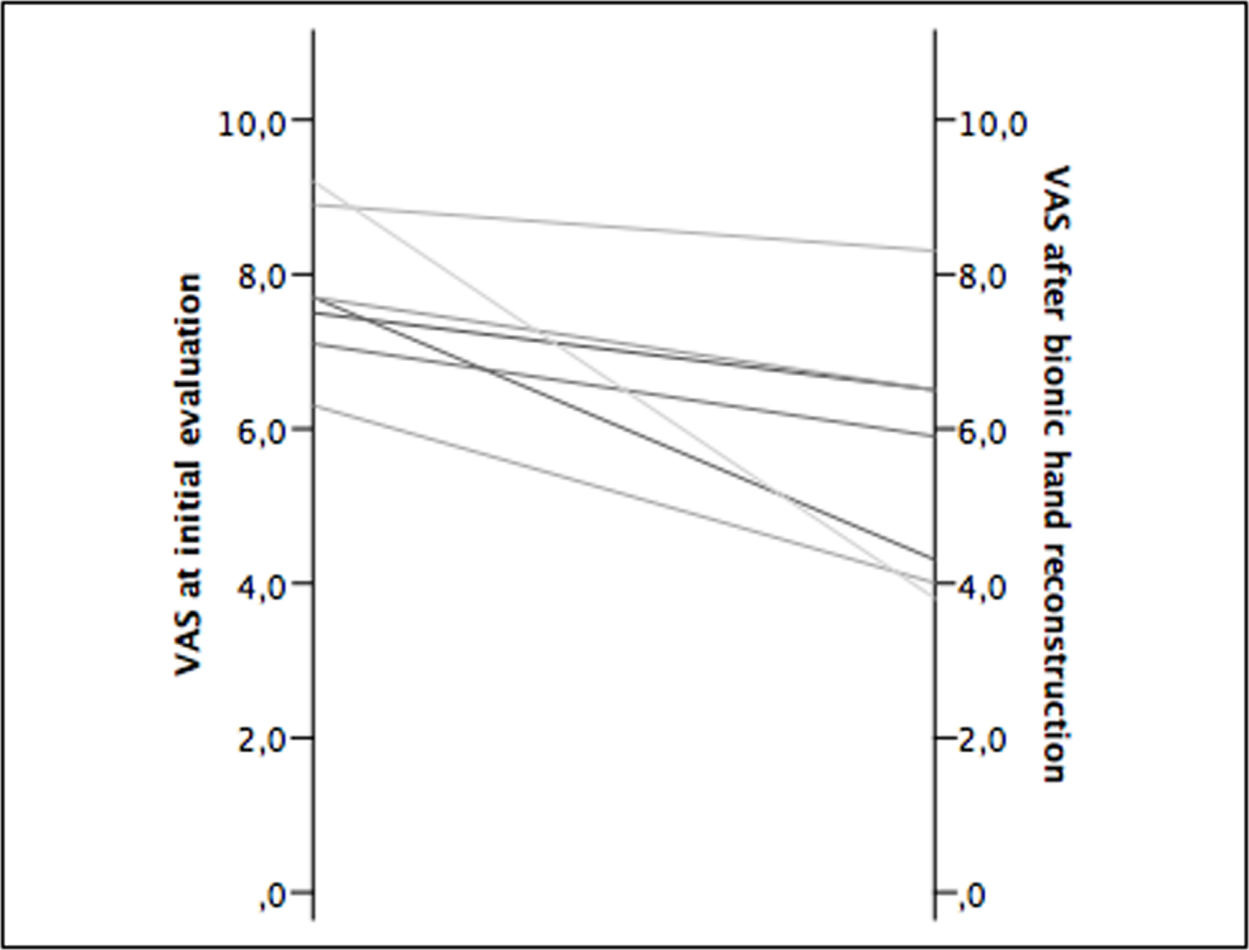
VAS scores for all patients. Pain was assessed at initial evaluation and after bionic reconstruction with a mean follow-up period of 10 ± 6 months.

VAS scores improved from 7.8 at initial evaluation to 5.6 after prosthetic hand replacement (p-value = 0.018) (Fig 3).

## Discussion

Although the main focus in brachial plexus repair is improvement in motor grade, a “good” motor result on paper is sometimes not of functional use to the patient as isolated functional gain in some cases might not improve the overall functionality in daily life [14]. As could be expected, all of our study patients reported of being functionally limited to a great extent due to either non-recovery or elbow-flexion recovery only. Except for Case 1, whose primary goal was the amputation itself, all other patients explicitly wished for functional improvement upon initial evaluation as most have seen the results of bionic reconstruction from media reports before consultation.

Psychological evaluation procedures have been described for a variety of surgical interventions [25],[26],[27]. For hand transplantation, a reconstructive treatment option for amputees without simultaneous damage to the nervous system [28], several evaluation instruments exist [29]. The elective amputation of an extremity–even if without functional use to the patient–also represents a major life-changing, and most importantly irreversible decision. We therefore decided to address similar domains as part of our psychological assessment procedure including history of psychiatric pathology, family support, chemical dependency history, knowledge about amputation and prosthetic rehabilitation, and levels of compliance [29]. With our qualitative approach using a semi-structured interview we aimed to unmask psychological symptoms resulting from the BPI within five different interview sections, A to E, which could eventually preclude successful bionic reconstruction and/or necessitate supportive psychological treatment before and during this procedure.

Interview section A addressed the patients’ experiences related to the accident that has caused global BPI including positive resources and coping strategies. Higher scores indicated inadequate processing and traumatic reactions related to the event. Psychological support was provided for patients with higher scores to prevent re-traumatization ensuing from an additional surgery (elective amputation) and associated hospitalization. Insufficient pain management right after the accident represented a stressor reported by 88% of patients, which should promote scientific progress regarding adequate pain medication regimes following a traumatic plexus injury involving nerve root avulsion.

The patients’ relationship to the injured arm and hand was addressed in interview section B. All except for one felt functionally limited to a great extent due to their disability. This attitude assured a patient’s conviction in favor of bionic reconstruction to improve function. Impaired bodily integrity and difficulties encountered from their social environment were reported by the majority of patients suggesting stigmatization and discrimination, as has been reported by others [14].

Interview section C covered the patients’ overall psychosocial status including prior psychiatric history, resources, coping strategies and substance abuse. All patients were able to describe positive resources during evaluation. Chronic deafferentation pain was perceived as dramatically debilitating by all patients. Substance abuse and/or addiction was defined as a strict contraindication for bionic reconstruction since adherence to instructions for prosthetic usage could not be expected.

Motivational aspects for an anticipated amputation were inquired in interview section D. Only one patient reached a score of 2 (maximum, 5) in that scale since functional improvement was not his primary intention for amputation. Some patients feared negative reactions from their social environment regarding their decision. In one case, psychological support was provided for the patient’s daughter, who was against an amputation. Her fears were discussed in an in-person interview with the same clinical psychologist responsible for conduction of the semi-structured interviews.

In interview section E expectations for prosthetic outcomes were discussed with the patients. The fitting of a prosthetic limb confronts patients with the irrevocable fact that they have lost a limb, must now adjust to wearing a prosthesis and must learn to be proficient in its use [30]. The highest score obtained in that scale was 1 (maximum, 5), which indicated that most patients had realistic expectations of prosthetic reconstruction after BPI with consequent reduced neural input and limited availability of electromyographic signals. Most stated that media reports about bionic hand reconstruction after BPI have attracted their interest for consultation. For some patients appointments with other BPI patients who had already undergone bionic reconstruction were organized to ensure realistic expectations about prospective prosthetic hand control.

Unrealistic expectations including fantasies of cyborg-like function via technological body enhancement in patients presenting with sufficient hand function need to be discussed critically. Bionic reconstruction only applies to patients who lack treatment alternatives and in whom primary and secondary biologic reconstructions have failed to restore a minimum of functional use. It is important to note that a prosthetic hand remains inferior to any biologically reconstructed function. Patients with delusive expectations of prosthetic function need to be informed adequately and candidacy for bionic reconstruction should be reconsidered.

The maximum total score of our semi-structured interview was 30, the highest score obtained in our study group was 10. A cut-off value for rejection of candidacy for bionic reconstruction had not been set, however, psychological “red flags” such as substance abuse, symptoms of a posttraumatic stress disorder, unrealistic expectations of prosthetic function, low adherence and compliance levels, etc. have been defined, which would preclude successful prosthetic usage. Additionally, the applied scoring system identified patients at-risk on an intra-individual basis and/or areas in which psychological support was necessary to improve patients’ quality of life as well as their understanding of bionic reconstruction.

Of the presented patients no one had been rejected for bionic reconstruction as the request of amputation was coherently linked to the wish of functional improvement by prosthetic hand replacement. A certain selection bias needs to be taken into account here, since patients may drop out at any time point and any step of the algorithm before the psychosocial assessment procedure is conducted (see Fig 2, e.g., we see patients who come to our center with severe nerve injuries for initial consultation, but are not interested in prosthetic reconstruction). The one patient who did not state that functional gain was his primary intention for amputation is the only one in our study population who does not wear his prosthesis on a regular basis, all others reported wearing times from 7 to 14 hours per day during regular post-interventional follow-up examinations. None of the patients have regretted their decision stating that the flail hand had only “been in their way” before amputation. At 10 months follow-up patient-reported questionnaires on quality of life and body image revealed positive effects of bionic reconstruction. Physical functioning as well as mental health improved significantly after the prosthesis had been incorporated into the user’s activities of daily living (Table 4). The significant improvement in the SF-36 Health Survey subscales “vitality” and “social functioning” was reflected by increased participation in social activities, increased self-sufficiency and reduced reliance on others as reported by the patients.

Discontent and insecurities regarding the physical appearance of a global brachial plexopathy can lead to high rates of social discomfort around strangers, feelings of unattractiveness and relationship interference [12]. During initial evaluation 50% of our patients reported difficulties with their bodily integrity and unpleasant social reactions to the appearance of their withered arm. In six of seven patients prosthetic hand replacement led to restoration of an intact body image by resolution of this negative body evaluation. When inquired about the grade of embodiment of the prosthetic hand during regular follow-up investigations, most patients reported that the prosthesis had become an integral part of their self-image using phrases like “For me this is not a mechatronic device. This is my new hand. I put it on right after waking up and mostly fall asleep at night having forgotten to take it off.” As is in agreement with statements like this, so far patients have not reported of any psychological burden associated with their decision of amputation or the completed prosthetic reconstruction. The weight of the prosthetic arm/hand has not been found debilitating after prosthetic training. During the first weeks of prosthetic usage, however, fatigue of the biceps muscle and/or the shoulder girdle have been reported after prolonged wearing which was accompanied by cumbersome prosthetic control and diminished dexterity. Upon regular usage, however, muscle strength steadily improved as did smooth control of the prosthesis. Patients have reported that technical maintenance of the prosthesis is considered a problem since giving the hand away for a few days interfered with their adaption to using two hands again.

Deafferentation pain is a common sequel of BPI including the avulsion of nerve roots [31],[32],[8]. Scar formation and hyperexcitability of injured neurons in the spinal cord segments of avulsed nerve roots are believed to cause this chronic, debilitating pain syndrome [33],[34]. Our psychosocial assessment procedure revealed chronic pain in all patients. The evaluation of the patient’s understanding, however, that the amputation itself would not relieve pain was of utmost importance to prevent unrealistic expectations. We knew from our experience with the first world-wide cohort of BPI patients treated with bionic reconstruction [18],[19] that prosthetic usage has the potential to reduce deafferentation pain. In our study population we found a significant improvement from VAS 7,8 to 5,6 after the prosthesis had been incorporated into the user’s activities of daily living. Interestingly, the patients who had used their prostheses for a longer period of time showed considerably greater pain reduction than the ones with short follow-up periods, with three patients reporting pain levels as little as VAS 4. Although the pain’s origin, which lies in the central nervous system [35], is left untreated we believe that functional restoration via bionic replacement provides a complex afferent input that overrides phantom pain, a phenomenon to which we refer as “functional re-afferentation”. This phenomenon does not come at once, but is a slow process that is mediated by regular usage of the prosthetic hand. Central reorganization, i.e. cortical remapping, is known to maintain deafferentation pain at a supraspinal level; the deafferented cortical area (= the hand region) is invaded by adjacent cortical areas, mostly the face [35]. Even if the bionic hand or arm will always remain an “assist” extremity, the regained bimanual dexterity represents an immense expansion of manual capacity [19]. We hypothesize that daily interaction of the prosthetic hand with the environment has the potential to reverse cortical reorganization [36] since the prosthetic hand may recover “intact” motor and somatosensory representation in the brain.

## Study limitations

This study had several limitations. In some interview sections data were likely limited by recall bias (i.e. inaccurate remembering of past events, emotions and thoughts) [12]. Our small sample size limits the generalizability of quantitative findings regarding quality of life, body image and VAS scores. However, considering the rare event of complete brachial plexus injury with lack of biological treatment alternatives, i.e. failed primary and secondary reconstructions, associated with the consistency of our qualitative findings (deafferentation pain, negative body evaluation, etc.), we feel that interviewing additional subjects will not significantly change our qualitative and quantitative findings. Of course a possible selection bias has to be taken into account. The psychosocial assessment procedure is only administered when the preceding steps of the treatment algorithm for bionic reconstruction have been successfully completed (see Fig 2). There are patients who initially show interest in the procedure but drop out before the administration of the psychosocial assessment which is obligatory before surgery (i.e., amputation). Hence, there is no comparison with patients who underwent initial preparations but then did not receive surgery.

Finally, the mean follow-up period of 10 months does not unmask long-term effects of bionic hand reconstruction on quality of life, body image evaluation and chronic pain.

## Conclusion

Bionic reconstruction improves physical functioning, mental health and social functioning, as well as chronic deafferentation pain and restores an intact self-image in patients with multiple root avulsions. Candidacy for bionic reconstruction needs to be considered critically. The presented psychosocial assessment procedures helps identify psychological issues, which could preclude or delay bionic reconstruction.

## Supporting information

S1 File Dataset for all patients. (XLSX)

## Abbreviations

BPI: brachial plexus injury
VAS: visual analogue scale
